# Galvanism in Uterine Hemorrhage

**Published:** 1847-12

**Authors:** 


ARTICLE II.

### GALVANISM IN UTERINE HEMORRHAGE.

We have received a communication from Dr. C. F. McNeill, of Concord, Ind., giving the result of his experience, in the application of Galvanism in exciting contractions of the uterus in profuse hemorrhage from that organ.— His manner of applying it is "to introduce the positive pole of a small portable electro-magnetic machine to the os uteri and place the negative pole upon the abdomen, immediately over the fundus of the uterus, and then regulate the electrical current as circumstances seem to require." He says, "the result is immediate and firm contraction of the uterus." As this fills the great indication of cure in cases of hemorrhage following delivery, we cheerfully comply with the request of our friend "by bringing the subject to the favorable notice of the profession." He also suggests the propriety of its application in labor.

The remedy was highly recommended by Dr. Radford, of England, about four years ago, but does not seem to have attracted much attention. He recommends it in hemorrhage before as well as after delivery of the child, and either before or after the delivery of the placenta.

From the powerful and certain influence of moderate currents of electricity, in producing muscular contraction, we would be led to the conclusion à priori that it would form one of our most reliable remedies in an affection that depends upon the relaxation of an organ so decidedly muscular in its character, as the uterus.

But when to its philosophy we have added the test of actual experience in its favor, without a single argument against it, is it not the part of wisdom, nay of duty, to use it?

The cases in which we conceive it more particularly applicable are those in which the hemorrhage follows delivery, as there can here be no danger of injuring the child, which is not certainly the case prior to delivery. In such cases we can see only one obstacle to its becoming our most common and most popular remedy, which is the difficulty of being always prepared with the battery. Certainly in a country practice it would be a great inconvenience for the physician to go prepared with the machine, especially as he might not find occasion to use it once in a long time, for the purpose, and unless he has it with him, the case will frequently terminate fatally, or be relieved by other remedies before he can procure it.

But in city and hospital practice, it must, if future observations corroborate those already made on its use, soon become the main dependence in the treatment of the cases of uterine hemorrhage above referred to, when the ordinary means fail.

There is one other thought in connexion with the subject, that occurs in consequence of the philosophy of the action of galvanism, in exciting muscular contraction, and our ability to apply it to any part we may desire, by placing the poles of the battery on opposite sides of the part through which we desire it to pass, which is that in cases of irregular contraction of the uterus, whether of the cervex, the hour-glass, or the contraction of the circular fibres without that of the longitudinal, from which forms fatal consequences often result, a current of electricity may excite the relaxed fibres to contraction, and produce the necessary equilibrium of action.

Again, we make the suggestion, that our professional brethren may, if occasion should offer, act on it, that when the placenta is retained by morbid adhesions to the uterus, violent contractions, such as may be excited by electricity, may cause its detachment and expulsion, which certainly would be far preferable to the painful and always dangerous operation of introducing the hand and separating it with the fingers. And in cases of abortion where the ovum is retained for a considerable time after its death, may not currents of electricity, passed through the uterus, promote its expulsion?

Another idea occurs, which is that in those cases of debility, always attended by a greater or less degree of relaxation of the uterus, where the lochial discharge continues too long after labor, the passage of currents of electricity may stimulate the organ to a more rigid condition, and thus check and arrest the discharge and afford relief.

And yet another idea;—is it not probable that currents of electricity passed through the uterus, energizing the muscular structure, and consequently, by continuity, the whole organ, may prove highly advantageous in chronic uterine leucorrhœa?

We hope these suggestions may prove useful in directing the attention of the profession to the application of galvanism in the various conditions suggested, and that we shall hear from those who may make a trial of it, by a report of their experience and observation for our pages. E.

